# Natural and Modified Zeolites as Adsorbents for Nitrogen and Phosphorus Control in Eutrophic Freshwater Bodies: A Comprehensive Review on Freshwater Applications of the Last 10 Years

**DOI:** 10.3390/ma18214870

**Published:** 2025-10-24

**Authors:** Irene Biliani, Ierotheos Zacharias

**Affiliations:** Laboratory of Environmental Engineering, Department of Civil Engineering, University of Patras, 26504 Patras, Greece; biliani.i@ac.upatras.gr

**Keywords:** zeolite, adsorption capacity, adsorption efficiency, ammonium, nitrogen, phosphorus, orthophosphate

## Abstract

Eutrophication of freshwater bodies is primarily caused by excessive nitrogen and phosphorus, resulting in significant environmental challenges, including harmful algal blooms and hypoxia. This review examines the potential for natural and modified zeolites to act as adsorbents and regulate nutrient concentrations in eutrophic freshwater ecosystems, excluding applications for wastewater or industrial water effluents. Natural zeolites are effective adsorbents of ammonium, whereas modified zeolites (with aluminum, iron, calcium, and many others) have been noted to have enhanced phosphate adsorption and a higher overall nutrient removal efficiency. The application of modified zeolites for controlling eutrophication in freshwater bodies has proven to have high efficiency in adsorbing nitrogen and phosphorus, resulting in reduced nutrient release from sediments and improved water quality in shallow lakes and reservoirs. This review describes the adsorption mechanisms and modification methods, with an appreciation for the multifunctional role of zeolites in nutrient immobilization and capping sediments. Finally, it presents the potential to use zeolite-based materials in eutrophic freshwater restoration through sustainable circular economy approaches. Zeolite materials present ample environmental applications for cost-effective and targeted mitigation approaches to freshwater eutrophication.

## 1. Introduction

Eutrophication is the excessive addition of nutrients, generally nitrogen (N) and phosphorus (P), to freshwater bodies, resulting in algae bloom, reduced water transparency, oxygen depletion, and fish deaths [1]. The increased nutrient loading, typically from human activities like farm and agricultural nutrient runoff or wastewater discharge [2], ultimately leads to water quality deterioration [3], significantly impacting the catchment [4] of the water body in general and on drinking water storage [5]. The Environmental Protection Agency (EPA) [6] and the European Union (EU) Water Framework Directive [7] outline eutrophication as a major threat to water quality and recommend strategies to reduce nutrients levels in the water. The nutrients that are the most critical are ammonium and phosphate [8] because they are readily taken up by algae, and are often the limiting nutrients for algal growth in freshwater bodies. Studies suggest that the thresholds of freshwater eutrophication are established when total nitrogen concentrations exceed approximately 0.8 mg/L (0.3 mg/L for ammonium nitrogen and 0.5 mg/L for nitrate nitrogen) and total phosphorus concentrations exceed approximately 0.05 mg/L [9,10]. However, these thresholds vary depending on the type of eutrophic water body (coastal, transitional, lake, river, sea, etc.) as well as each water body’s reference conditions as described in the WFD [7]. Therefore, measures are required to simultaneously reduce both nutrients (N and P) and mitigate eutrophication, ultimately restoring water bodies’ quality status [11].

The restoration of eutrophic waters is now an essential and urgent global priority that cannot be ignored. A wide range of procedures and practices are proposed by the scientific community to control nutrient concentrations in water bodies by either retaining nutrients in bottom sediments or permanently removing them from the eutrophic ecosystem [12]. Eutrophic restoration practices can be classified as physical, chemical, biological, combined, and innovative techniques [13]. The application of clay-based adsorbent materials is a chemical adsorption technique, which is widely proposed by the scientific community as an effective method to address the phenomenon of eutrophication [14].

Zeolite materials have proven to be highly effective [14] in mitigating eutrophication, based on the specific and complementary removal capabilities of both nitrogen and phosphorus. Natural zeolite is a mineral material distinguished by its porous structure. It consists mainly of clay silicates and contains primarily sodium (Na), potassium (K), and calcium (Ca) [15], presenting a high specific surface area and thermal stability [16]. Natural zeolites, especially clinoptilolite-rich zeolites [17], are a suitable medium for the removal of ammonium ions, owing to their inherent cation exchange capacity (CEC) [15] and ability to preferentially associate with ammonium over other competing cations (Na^+^ and Ca^2+^) commonly present in freshwater. The removal of phosphorus species, namely phosphate (PO_4_^3−^-P), is limited by natural zeolites’ inherent inability to adsorb phosphorus species due to electrostatic repulsion between the negatively charged framework of the zeolite [18] and the negatively charged phosphate species. Thus, modified zeolites, which have a greater capacity for phosphate removal than their natural forms, are able to achieve simultaneous nutrient control to effectively mitigate eutrophication. For example, once the zeolite surface has been impregnated with cations [19] of another metal (Fe^3+^, La^3+^) [20], the surface charge is altered and allows for new sites of adsorption on the zeolite surface when reacting with anions such as PO_4_^3−^-P (either by ligand exchange [21] or surface complexation [22]). Therefore, modifying and then selecting a modified zeolite represents an important step to addressing the dual nutrient problem of N and P in eutrophic waters.

In parallel, incorporating circular economy practices in the treatment of eutrophication using zeolites is crucial for a more sustainable and resilient aquatic ecosystem. Natural and modified zeolites have the potential to be regenerated and reused numerous times. This approach would benefit the aquatic water body by avoiding overloading the benthic zone with residual zeolite-based material by removing the zeolites from the water body. These approaches exploit either the iron-modified zeolites’ paramagnetic characteristics [23] or introduce innovative methods such as teabag applications [24]. Up until recently, only preliminary studies have been conducted on circular economy approaches with zeolite-based materials [25] in regard to the mitigation of eutrophication.

The goal of this review is to create a connection between fundamental lab research on zeolite adsorption and its real-world applications in preventing/treating eutrophication of natural waterbodies. Although the scientific literature contains an ample number of important lab-based studies on zeolite materials and studies in a controlled mesocosm environment, there is a clear gap in large-scale treatment recommendations regarding zeolites’ modification protocol, particle size, pH range, and dosing/contact time.

Also, despite the increasing number of zeolite-based nutrient removal studies, there is a clear gap in zeolite applications for eutrophic freshwater body systems. Previous reviews generally addressed zeolites in either wastewater treatment or heavy metal adsorption, clearly delineating a knowledge gap on their use in the presence of nutrients. In addition, adsorption capacities are usually defined in non-standard units, and the methods of modification are described without coherent integration. This review addresses these issues by (i) norming adsorption definitions, (ii) providing meta-analysis on modification methods, and (iii) identifying knowledge gaps and suggesting research priorities. This study reviews the current applications of zeolite from 2015 to 2025, examines available removal efficiencies and capacities, and identifies the mechanisms of sorption implemented.

## 2. Materials and Methods

### 2.1. Data Sources and Inclusion/Exclusion Criteria

A systematic literature review was performed to synthesize existing knowledge on the use of natural and modified zeolites for the mitigation of eutrophication in freshwater systems. The literature search was carried out using the Scopus, Science Direct, and Web of Science academic databases and included peer-review articles without conference proceedings, and technical reports. The search date ranged from 2015 to the present (2025) in effort to include recent advances in zeolite applications in environmental sciences. The software Grammarly (v 1.2.113.1522) was used for minor English editing.

**Figure 1:**
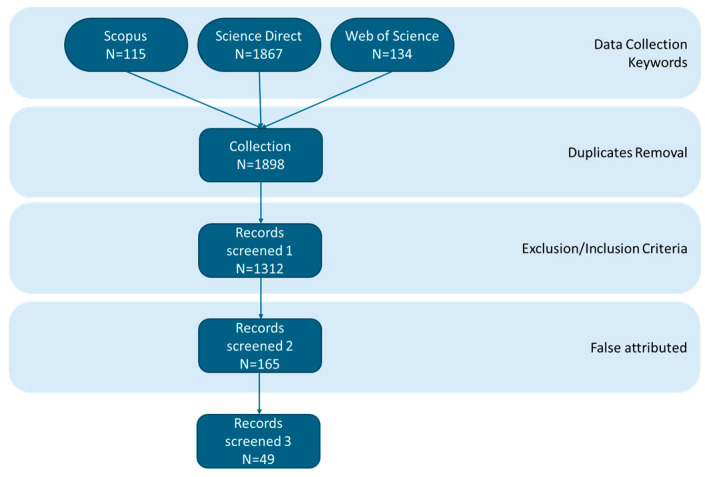
The PRISMA flow chart illustrates the methodology conducted to conduct the literature review of this study.

The scientific literature search was conducted under the PRISMA 2020 guidelines and included the following keywords: “eutrophication”, “adsorption” and “zeolite”. After removing duplicates from the dataset, the inclusion criteria were set as “natural zeolite”, “modified zeolite”, “ammonium”, “ammonia”, “nitrogen”, “phosphorus”, “phosphate”, “orthophosphate”, “water”, “freshwater”, “literature review”, “adsorption capacity”, and “adsorption efficiency”. Studies with the following keywords were excluded from the analysis: “wastewater”, “waste management”, “waste treatment”, “waste component”, “blackwater”, “industrial”, and “industrialization”. Search queries were later refined to obtain the most relevant studies. For instance, studies where the primary adsorbent medium was not a zeolite (e.g., “biochar”, “activated carbon”) were excluded, even if a zeolite was used as a minor component or a reference. Figure 1 illustrates the methodology conducted for this scientific literature review.

**Figure 2:**
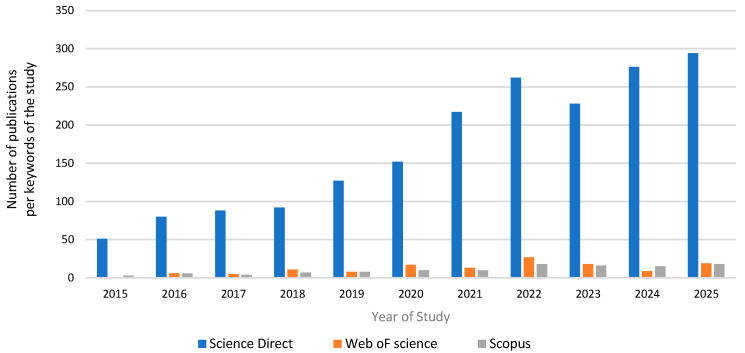
Bibliometric analysis of the keywords “eutrophication”, “adsorption”, and “zeolite” during the period 2015–2025 from the scientific datasets Science Direct, Web of Science, and Scopus.

The bibliometric analysis of the selected dataset reveals a growing interest in the area of eutrophication control with the application of zeolite as a primary adsorbent. Figure 2 denotes that during the years of study 2015–present, the number of publications has increased markedly. This trend reflects the emergence of the zeolite-based adsorbents for controlling eutrophication as a priority research agenda from the scientific community as a sustainable water management approach.

### 2.2. Data Manipulation

Up until today, there is no common representation of zeolites’ adsorption capacities in the vast scientific literature. Nonetheless, an accurate review and comparison of the adsorption capacities of the materials introduced lies in the homogeneous representation of the results. This review addresses the challenge mentioned above, after careful conversion of the results of each paper in the following units: mg of NH_4_^+^-N/g and mg of PO_4_^3−^-P/g.

The molecular weight of the ammonium ion (NH_4_^+^) is almost 18 g/mol, whereas the molecular weight of the ammonium ion in the form of nitrogen is 14 g/mol. Therefore, the conversion ratio from NH_4_^+^ to NH_4_^+^-N is 14/18, equal to 0.7778. Therefore, the required conversion is the one presented in Equation (1):

(1) $${NH}_{4}^{+}-N={0.778\;NH}_{4}^{+}\;$$

Similarly, the molecular weight of the orthophosphate ion (PO_4_^3−^) is almost 95 g/mol, whereas the molecular weight of the orthophosphate ion in the form of phosphate is 31 g/mol. Therefore, the conversion ratio from PO_4_^3−^ to PO_4_^3−^-P is 31/95, equal to 0.3263. Therefore, the required conversion is the one presented in Equation (2):

(2) $${PO}_{4}^{3-}-P={0.3263\;PO}_{4}^{3-}\;$$

## 3. The Modification Procedures and the Adsorption Mechanism

### 3.1. Different Modifications of Zeolites

**Figure 3:**
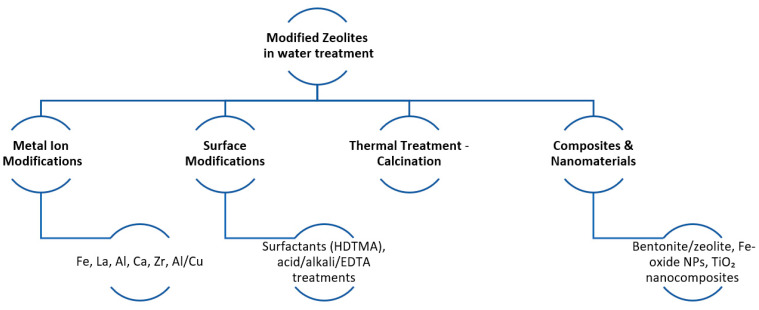
Schematic diagram of the modification processes that zeolite undergoes for water treatment.

Modifications of natural zeolites aim primarily at increasing adsorption of anions such as orthophosphate ions. Typical modifications are made to improve the surface charge, the pore structure, and the availability of adsorption sites of the zeolite. The main modifications of zeolites that have been implemented over the last 10 years as reviewed in this study can be classified into the following categories: metal ion modifications, surface modifications, calcinations, and composite and nanostructure zeolite incorporation (illustrated in Figure 3).

Metal-ion modifications have often been used to enhance phosphate adsorption. For instance, ferric-modified zeolites usually have a higher capacity to sorb phosphate from aqueous solutions because iron hydroxides that are bound to the surface provide more adsorption sites [26]. However, research shows that ion exchange can, at the same time, reduce the ammonium adsorption capacity, since the new positively charged iron oxides may be repelling ammonium ions with their electrostatic repulsion, or they may be blinding the negatively charged surface area by causing dealumination [27,28]. Lanthanum (La) is a practical modification example due to its affinity for phosphate, as La forms strong complexes and precipitates in aqueous and lake waters [29,30].

Aluminum (Al) and calcium (Ca) modifications have proven very successful in providing phosphate removal capacities. For example, aluminum-modified zeolites (AMZs) fabricated from lake sediments provide higher adsorptive capacities with phosphate because the phosphate is being bound via hydrous metal oxides, while the zeolite itself can continue to add abstractions via negative sites of internal structure and cation exchange [27,31]. Pretreatment with calcium creates available Ca^2+^ ions for the precipitation of Ca phosphate and has been found to have the potential to increase phosphate loadings, with a negative consequence for ammonium exchange capacity [27]. For Al/Cu-modified combined zeolites from Ethiopia [32], the composited zeolites achieved a high removal percentage for phosphate when the phosphate and dissolved matter were facilitated through chemisorption via ion exchange, precipitation, and ligand exchange, while providing more sites for adsorption. Also, zirconium (Zr)-modified zeolites (ZrMZ) constructed from fly ash were most effective in adsorbing phosphate from lake water once they became activated for phosphate because they provided accessible and available adsorption sites [33]. Finally, modifications of natural zeolites that enhance their ammonium removal efficiency often include impregnation with NaCl, NaOH, and Na citrate solutions [34], since Na^+^ has a greater capacity for ion exchange than other cations (K^+^, Mg^2+^, Ca^2+^, and Cu^2+^) [35]. Studies that employed the impregnation of zeolite with NaNO_3_ solutions, and later calcination of the zeolite, led to increased ammonium removal equal to 40% compared with the ammonium removal of natural zeolite [35].

Surface modification techniques such as using Ethylenediaminetetraacetic Acid (EDTA) can increase ammonium adsorption. Surface modification with EDTA will introduce new functional groups that will alter the surface properties and the pore structure of the zeolite, revealing active sites and increasing selectivity via hydrogen bonding and electrostatic interactions [36]. Other methods, such as acid and alkali treatments, can be controlled for a desired Si/Al ratio, which can affect zeolite hydrophilicity/hydrophobicity and, thus, their respective adsorption capacity [18,37]. Acid and alkali treatments have the ability to increase surface area, mesopore volume, and surface area coverage, but the amount of acid or alkali used needs to be controlled, as the previously mentioned treatment strategies can destroy zeolite structure if used incorrectly [18,37].

Another well-presented methodology is thermal treatment or calcination, which involves exchanging water using elevated temperatures, which increases the specific surface area of the sample material while additionally promoting the diffusion of ammonium into the pores, increasing the potential for ammonium exchange [35].

Finally, a newer and more promising application involves the production of composite materials such as bentonite/zeolite (BE/ZP), which increases both ammonium and phosphate removal efficiencies due to enhanced porous properties, increased surface area, and increasing potential for ion exchange, enabling physisorption by coulombic attractive forces and chemical interactions [38]. Other applications include the incorporation of green, hydrothermally synthesized iron oxide nanoparticles, which were dispersed onto zeolite to concurrently remove ammonia and phosphate (EL-MNP@zeolite) [8]. Zeolite acted as a carrier for the nanoparticles, resisting agglomeration, while phosphate removal occurred by chemical adsorption of phosphate onto the zeolite through interaction with the Fe-OH bonds, and ammonium was removed by physical adsorption via electrostatic attraction [19]. Finally, TiO_2_/zeolite nanocomposites have been shown to very effectively remove both ions through chemisorption, where the adsorption of phosphate was often much higher that of ammonium [39].

The analysis of the recent literature indicates there are a few important patterns. Natural zeolites, without any modification, are being replaced by chemically or thermally modified materials, which generally demonstrate enhanced selectivity and adsorption capacity for both ammonium and phosphate [14,40]. Furthermore, hybrid [41,42] and composite zeolites are gaining interest and are part of an overall movement toward multifunctional adsorbents. An in-depth understanding of the mechanisms that govern each water body will allow practitioners to select the modified zeolites that are appropriate to use in each freshwater body for eutrophication control.

### 3.2. Adsorption Mechanism

Zeolites consist of an elementary structure of an aluminosilicate framework whose the tetrahedral arrangement has a silicon ion (Si^4+^) and an aluminum ion (Al^3+^) surrounded by four oxygen anions (O^2−^) [22]. Each oxygen ion occurring in Si-O and Al-O bonds connects to two cations and is shared between two tetrahedron structures [21]. This results in a tetravalent electroneutral Si and a trivalent negatively charged Al. The negative charge and the pores of zeolites can be filled with group IA or IIA metal ions and water molecules as represented through the general formula Mx/n {(Al_2_O)x (SiO_2_)y zH_2_O} [43], where M is an alkali or alkaline earth cation, n is the valence of the cations, z is the number of water molecules per unit cell, and x and y are the total numbers of tetrahedra per unit cell. As a result, the adsorption mechanism of zeolites may differ between natural zeolites and modified zeolites, due to selectivity at the exchange sites of the zeolite system, the cation exchange capacity (CEC), the surface charge, and the pore structure of the modification that has been performed.

Zeolites’ primary mechanism of adsorbing ammonium ions is through ion exchange [44,45]. The NH_4_^+^ takes the place of an exchangeable cation (e.g., Na^+^, Ca^2+^, K^+^, or Mg^2+^) in the zeolite framework [46]. Natural zeolites have been widely used for the adsorption of ammonium ions, showing excellent ammonium adsorption efficiencies [47,48,49,50]. Recent studies declare that zeolites’ ion exchange selectivity governs phase selection [51] and that the selectivity series (for monovalent metals) differs depending on the synthesis of the zeolite [52]. Other studies suggest a different ammonium removal pathway by tracking Ca^2+^ and Al(OH)_4_ concentrations in an aqueous solution [18,53].

Parallel to ion exchange, the negatively charged zeolite surface has a strong attraction for positively charged ammonium ions [54], a phenomenon recently identified as electrostatic attraction. Electrostatic attraction can play an essential role in amorphous zeolitic materials, especially during the initial stages of adsorption, and in the pores of zeolite frameworks.

Studies of natural zeolites indicate that chemisorption, which is defined as the sharing or exchange of electrons between ammonium ions and the zeolite surface, is also a separate mechanism in the adsorption of ammonium [54]. This results in the development of hydrogen bonding between ammonium ions and the oxygen atoms in the zeolite framework, which secures the binding of the ammonium ions with the zeolite surface. For example, Mg-ZIF nanosheets showed ammonium adsorption derived from monolayer chemisorption by electrostatic attraction and hydrogen bonding [55].

**Figure 4:**
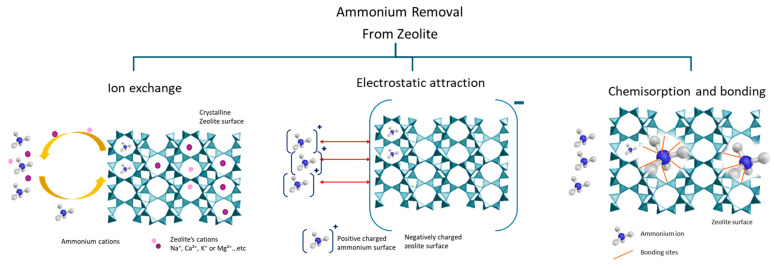
Schematic presentation of ammonium adsorption mechanisms by zeolite composites.

The ammonium adsorption capacity of natural zeolites varies based on the Si/Al ratio, mesoporous volume, specific surface area, and cation exchange capacity. The presence of larger mesopores will aid the transport of molecules found on active sites that may be bound more tightly to micropores [35]. Figure 4 illustrates different ammonium ion adsorption mechanisms. Depending on the modification of the zeolite, different mechanisms take place and govern the sorption process.

Similarly, for orthophosphate ions, depending on the modification the zeolite has undergone, a different adsorption mechanism takes place. Generally speaking, phosphate adsorption on zeolites, due to the negative surface charge of natural zeolites, is more complex than that of ammonium ions, because they typically have little, if any, affinity for anionic species [31,50]. Therefore, modification is either through precipitation, ligand exchange, or adsorption onto surfaces based on Al or metal oxides.

The phosphate ions from metal ion-modified zeolites, such as Ca^2+^, La^3+^, Fe^3+^, Al^3+^, etc., usually tend to occur through precipitation. Ca-modified zeolites would exchange calcium ions with sodium ions in solution for phosphate species in the solution to precipitate Ca phosphate (CaP) structures [56]. In synthetic aquatic solutions with the dual presence of ammonium and orthophosphate ions, the CaP precipitation would be increased by liberating additional Ca ions from the zeolite.

In the case of modified zeolites, whose surface charge can be altered to become more positive, or for modified zeolites that contain metal oxides/hydroxides that provide positively charged sites, electrostatic attraction would occur between the negatively charged phosphate species and positive surface sites [57]. The formation of surface complexes allows the storage of orthophosphate ions in the pores of the modified zeolite.

**Figure 5:**
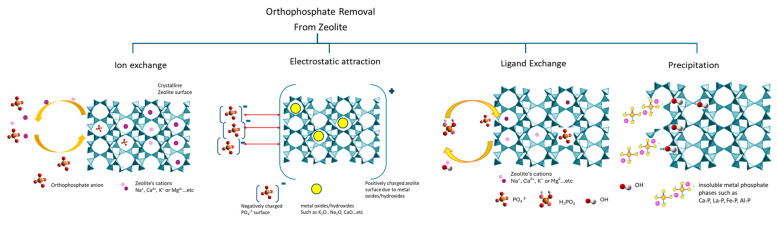
Schematic presentation of orthophosphate adsorption mechanisms by zeolite composites.

Currently, the most favored mechanism for phosphate ion (H_2_PO_4_^−^, HPO_4_^2−^, PO_4_^3−^) adsorption onto metal-modified zeolites (e.g., lanthanum, iron, aluminum, or zirconium) is the ligand, which is determined from the selection of the phosphate solution that is added to the adsorbent framework [41]. In this case, the phosphate ions displace hydroxyl (OH^−^) groups that are found on the metal oxide surfaces of the modified zeolite and create stable inner-sphere structures (e.g., La-OPO, Fe-O-P), therefore effectively “caging” phosphate to the adsorbent [41]. Figure 5 is a visual presentation of the adsorption mechanism of orthophosphate ions from different zeolites.

Both the ammonium and the orthophosphate adsorption mechanisms were significantly affected by the contact time to reach equilibrium, the initial concentration of the pollutants, the dosage of the adsorbent, and the presence of competing ions in freshwater conditions by all these modifiers. Studies of zeolite materials are well described by pseudo-second-order kinetic models, indicating a chemisorption mechanism for both ammonium and orthophosphate ion adsorption. Also, Weber–Morris kinetic results of zeolite materials prove that the mechanism of adsorption is completed in multiple phases, not just the rate-limiting phase.

## 4. Discussion

### 4.1. Comparison of Zeolite Adsorption Capacity Related to Zeolites’ Different Origins

There are 67 species of natural zeolites that have been identified to date [58]. Clinoptilolite, mordenite, analcime, chabazite, fillipsitis, erionite, evlanditis, natrolithos, and stilvitis are natural zeolites that appear as imprints or deposits. China is the largest producer of natural zeolites (320,000 t in 2018), followed by Korea, New Zealand, the United States, Turkey, and Cuba [59]. A simple geographical classification of zeolites consists of European, Asian, African, and North American zeolites.

A comparative analysis of Greek, Slovakian, and Bulgarian clinoptilolite zeolites showed varying efficiencies [50] among the European zeolites. Researchers found that Greece and Slovakia generally showed higher ammonium and orthophosphate adsorptions compared to Bulgaria. The adsorption capacity of natural Greek and Slovakian zeolite reached 35 mg/g for ammonium ions and around 18 mg/g for orthophosphate ions [50]. In the same study, ZeoPhos GR achieved 39.55 mg/g NH_4_^+^-N capacities, and ZeoPhos SL achieved a balanced dual functionality of 36.87 mg/g NH_4_^+^-N and 36.88 mg/g PO_4_^3−^-P [50]. In another study, the natural Romanian clinoptilolite was able to reach around 13 mg/g ammonium adsorption capacity under elevated pH and temperature conditions [60].

Numerous studies present Chinese and Turkish natural zeolites, which adequately represent Asian zeolites. In particular, heulandite-derived zeolites have shown admirable ammonium adsorption ability, with some showing a maximum ammonium adsorption capacity of 26.94 mg/g at high concentrations [61]. The ammonium removal was also enhanced beyond zeolite modification with sodium nitrate calcination, which showed 81.68% ammonium removal [35]. In addition, zeolites produced from lake sediments in China also showed all-around high ammonium and phosphate capacities [31].

The initial examples above highlight an essential aspect of zeolite performance: Meaningful variances exist in zeolite function due to distinct geographical sources, degree, mineralogical composition, and geological formations. These 10 zeolites, from different areas of China, show that significant variance in baseline phosphate adsorption capacity exists (from 9.3 mg/g for Wuxi to 16.3 mg/g for Wuhan zeolite) [31]. It is possible that the differences in molecular ordering and structural complexity are partly a result of differences in Si/Al ratios, cation composition, and structural defects from geographical sources. Modification with aluminum also significantly enhances the ability for phosphate removal in all Chinese sources; however, the magnitude of its enhancement spans a wide range of fold change of improvement, with Binzhou zeolite having the highest improvement factor of 3.2.

Fewer studies have been conducted in the last 10 years on African zeolites for the control of eutrophication in freshwater bodies. Modified zeolites from Ethiopia (modified the Al/Cu way) from Hamusit were shown to successfully removed 99.32% of phosphate with acidity [32]. Finally, another example from British Columbia, Canada, represents one of the North American zeolites. Modification with zirconium demonstrated improved phosphate adsorption from aqueous solutions, with comparable results with magnesium–ammonium-modified zeolite, which was not as effective as initially expected [62,63].

### 4.2. Comparison of Ammonium and Orthophosphate Performance per Particle Size

Zeolite particle size has an important effect on its properties as an adsorbent. In the studies in our literature review from the past 10 years, scientific publications largely relied on zeolite modifications and chemical composition, and investigated the adsorption removal efficiencies, while only three studies compared zeolite particle size and had a sole interest in the adsorption of ammonium ions. Table 1 presents the analyzed information on zeolite materials with particle sizes spanning over five size categories, from 0.0048 mm to over 32 mm. The materials include natural zeolites from Romania [60], processed clinoptilolite (CLP85+) [49], micro-sized zeolite particles [64], and polymer–alginate hydrogel beads containing entrapped zeolite particles [64]. Ammonium adsorption capacities range from 10.46 mg/g to 60.6 mg/g, demonstrating significant variation based on material properties and particle size.

The improvements in ammonium removal efficiencies can be explained by the amount of specific surface area available with a smaller zeolite particle size, which provides both more opportunities for external surface adsorption and cationic exchange capacity sites. Also, most of the studies with smaller particles witnessed faster kinetics, along with increasing capacities, with other studies estimating that total ammonium sorption capacities for natural zeolite granules (e.g., 1–32 mm) were limited to a portion of the number of exchangeable sites, which were contained within the zeolite structures themselves and not limited to just those that were related to the investigated surface area. In addition, it is possible that each of the zeolite sizes in the studies had similar efficiencies rates (97–98%) and no efficiencies influenced by grain size [49]. Even in those comparisons, and as an aside, initial ammonium adsorption is generally slightly more rapid for the “finest” grain material [40].

**Figure 6:**
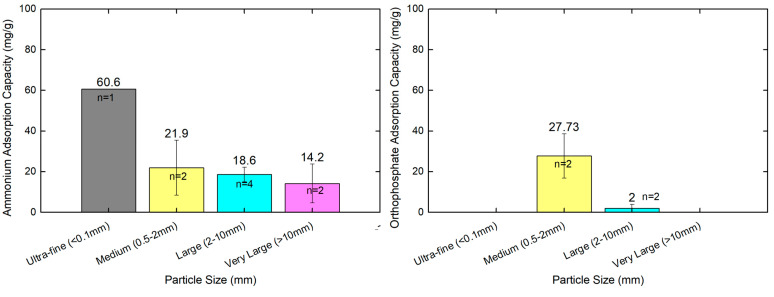
Comparison analysis of particle size of different zeolites and their relevant ammonium and orthophosphate adsorption capacity. Sources: [18,49,50,52,60,61,64]. Visualization was performed in Origin Lab (version 9).

In the scientific literature in this review from the past 10 years, no comparisons relating the zeolites’ particle size with the adsorption capacity or performance of orthophosphate or phosphate ions in general. Most, if not all, of the scientific papers offered the best or most notable potential remedies for increasing orthophosphate removal by modifying zeolite materials. One example, a modified novel zeolite material called ZeoPhos, had increased it’s orthophosphate removal efficiencies (up to 70% over its natural zeolite 55%), due to the incorporated iron, calcium, and humic acid ions [40]. In general, studies basically investigated orthophosphate adsorption performance differences and attributed them in modification protocol or zeolite variations [63]. Consequently, while zeolite modifications aiming to enhance the capacity for orthophosphate capture are still important, the literature does not provide evidence that effectively eliminates the competition among different zeolite particle sizes (and their variations) for orthophosphate adsorption capacity. Figure 6 attempts to visualize the results obtained from different publications comparing the particle size for ammonium and orthophosphate ion adsorption capacities.

### 4.3. Comparison of Natural and Modified Zeolite Adsorption Efficiency Compared to the pH Levels

The pH of a water body significantly affects the adsorption behavior of zeolite materials regarding the performance of ammonium or orthophosphate ions. The adsorption behavior of ammonium ions is generally enhanced in the neutral to moderately basic pH range. Studies show that significant ammonium adsorption by natural clinoptilolite zeolite often occurs between pH 6 and 8 [60,65]. At low pH levels, it is generally noted that ammonium adsorption efficiency is reduced, mainly due to increased competition with H^+^ ions in the solution. The mechanism is simple: Ammonium possesses a positive charge, whereas zeolite surfaces exhibit enhanced negative charges as pH rises above neutral levels. Consequently, as the pH of the solution approaches neutral or exceeds it, zeolite surfaces will acquire a greater negative charge due to the deprotonation of surface functional groups, enabling then to adsorb more soluble ammonium ions. A crucial finding in numerous investigations is that when the pH exceeds 9–10, ammonium ions can dissociate and commence transformation into neutral gaseous ammonia (NH_3_) [66]. This reduced uptake of ammonium ions occurs because negatively charged zeolite surfaces are less effective in the uptake of neutral ammonia compared to positively charged ammonium ions. Modified zeolites behave in the same manner in terms of ammonium uptake potential, with ammonium uptake efficiency maxima hypothetically developing in the pH 6–8 range. Chitosan-modified zeolites (CTS-ZMS [5]) present the optimum protonation state of chitosan functional groups and the speciation of ammonia, maximizing both ion exchange (Na^+^ ↔ NH_4_^+^) and heterogeneous adsorption mechanisms at a pH level equal to 6.5. In addition, other researchers describe ammonium ion removal as decreasing substantially “faster and faster” at pH values greater than 10 in the case of lanthanum-modified zeolite-type material [67].

Orthophosphate ion adsorption behavior tends to show different pH dependencies, primarily related to the speciation of the phosphate ion and the surface charge characteristics of the adsorbing material. Orthophosphate is taken into the adsorbing material in reduced amounts at high pH [30,68]. Higher pH causes phosphate species to convert to negative species (HPO_4_^2−^ and PO_4_^3−^), which are even more negatively charged and are repelling from a similarly negatively charged surface. Peak removal of orthophosphate in most modified zeolite compounds is often found under acidic to mildly acidic conditions, predominantly within the pH range of 2 to 7 [32] or 3 to 7 [69], due to the predominant phosphate species being monovalent H_2_PO_4_^−^. Within this range, lanthanum-modified zeolites exhibit a strong attraction for monovalent phosphate species (H_2_PO_4_^−^), since strong bonds of the La(OH)_2_^+^ structure are developed, which may augment the electrostatic attraction between the adsorbent surface and the phosphate, owing to the readily displaced (OH)_2_^+^ structures [69]. In parallel, Al/Cu-modified zeolites from Ethiopia demonstrated 99.32% phosphate adsorption at pH 2 [32], while zirconium-modified zeolites achieved optimum phosphate adsorption efficiency at pH levels equal to 7 [33]. On the contrary, other studies show that natural zeolite exhibits greater efficacy in phosphate removal within an alkaline pH range [70].

In general, other operational parameters include adsorbent dose, initial concentration, and temperature. Increasing the initial concentration decreases the zeolites’ achieved removal efficiency due to site saturation [71]. Studies show that lower temperatures favor zeolites’ ammonium adsorption [36], while PO_4_^3−^ adsorption can be temperature-insensitive [70].

### 4.4. Comparison of Zeolites’ Ammonium and Orthophosphate Adsorption Capacity

Zeolites’ established performance hierarchy reveals substantial variations contingent upon the identity and extent of structural modifications. Comparative results of ammonium and orthophosphate ion adsorption capacity are presented in Table 2. Metal-modified zeolites have the most diversity of applications for nutrient removal, and lanthanum-modified zeolites have been reported to have impressive phosphate adsorption capacities of 52.25 mg/g [30,72]. The ability of lanthanum to improve zeolite performance for phosphate is based on its binding constant to phosphate species and the formation of stable inner-sphere complexes that may improve the selectivity of the adsorbent [30,31]. In addition, aluminum-modified zeolites, collected from several sources across China, show that phosphate removal was also improved, with a significant improvement from Binzhou aluminum-modified zeolite-38.9 mg/g [31] (a threefold increase compared to unmodified natural zeolite) [55], setting a new benchmark for nitrogen removal applications. This enhanced capacity likely results from the combination of zeolite ion-exchanging characteristics and the high surface area and adjustable pore structure of novel metal–organic framework materials. The TiO_2_/zeolite nanocomposite material demonstrateda notable dual-functionality (adsoption of both 3.75 mg/g for NH_4_^+^-N and 38.63 mg/g for PO_4_^3−^-P) [39]. Additional work on modified zeolites, specifically lanthanum-modified zeolite (LMZ), has documented high efficiencies (78% for ammonium and 86.9% for phosphate [73]) and shows potential for transforming sediments from nutrient sources into nutrient sinks.

Nano-zeolite-based materials have been evaluated in laboratory and field trials. These multifunctional geoengineering materials based on zeolite and silica do not just adsorb nutrients, but also provide oxygen nanobubbles that can reverse sediment hypoxia and increase the reduction of sediment nutrient fluxes [74,75]. The results have been the promotion of beneficial microbial activity while improving water quality.

The variety in performance across modification schemes correlates with the performance variation based on the fundamental differences in adsorption mechanisms, and thus, active site chemistry. Iron-modified zeolites show moderate performance, with Fe (III)-modified zeolites showing 27 mg/g NH_4_^+^-N capacity, and iron can be delivered through several methods to have similar and moderate phosphate removal capacity at 0.159–0.186 mg/g [28]. These performance differences for iron-modified zeolites compared to lanthanum-modified zeolites can be attributed to the importance of the metal–phosphate interaction driving selective adsorption behavior.

Natural zeolites’ adsorption capacity results deviate from other zeolites, with a performance range of less than 1 mg/g capacity of nitrogen and phosphorus species [49,76], whereas other zeolites present increased ammonium adsorption capacity [49]. The significant performance shift in zeolites achieved through targeted modifications suggests it is rational to infer that rational design methods could be introduced to achieve the next generation of zeolite materials.

The dual functionality of zeolite materials enables the simultaneous removal of nitrogen and phosphorus, introducing a novel dimension to treatment technology. ZeoPhos SL balanced performance remarkably, with nearly identical phosphate and nitrogen removal capacities on average [50], while TiO_2_/zeolite nanocomposites achieved selective phosphate removal with moderate nitrogen activity [39]. The Ca/Fe-layered double-hydroxide composite demonstrated a phosphorous removal capacity of 46.8 mg/g [77], demonstrating a level of launcher capacity with targeted functionality.

The range of performance benchmarks for lanthanum-modified zeolites was consistent across studies, with a removal capacity for the LAH zeolite with 5.3% La of 76.3 mg/g [78]. Moreover, lanthanum-modified zeolite offers tradeoffs in selectivity for phosphate precipitants, which are effective at co-removing nitrogen, exemplifying the targeted optimization needed to manage dual-purpose systems with objective cost consideration.

However, zeolites’ ammonium and orthophosphate adsorption capacities deviate in aging experimental studies or long-term in situ or pilot applications. In long-term environmental applications, modified zeolites can release ammonium ions. In a 210-day lake sediment remediation study, ammonium fluxes across the sediment–water interface increased over control values during the final stages, indicating eventual release due to saturation of the material [79]. In contrast to ammonium, orthophosphate is more strongly attached to the zeolite’s framework. Studies simulating long-term exposure to challenging lake conditions (high pH and dissolved organic matter presence) reported minimal release, with desorption often quantified as less than 5% for Zr-modified zeolite [33].

Performance profiles overall provide the potential for key application niches for complicated zeolite modification schemes. High-capacity materials like Mg-ZIF nanosheets represent a key application niche for concentrated ammonium removal. Meanwhile, lanthanum-modified zeolites have advantages for selective phosphate removal from wastewater streams. The balanced but moderate performance of ZeoPhos materials represents an adequate and economically attractive option for general-purpose applications for nutrient removal, while clarifying that their dual functionality is further rationale for considering these devices ahead of single-nutrient devices.

**Figure 7:**
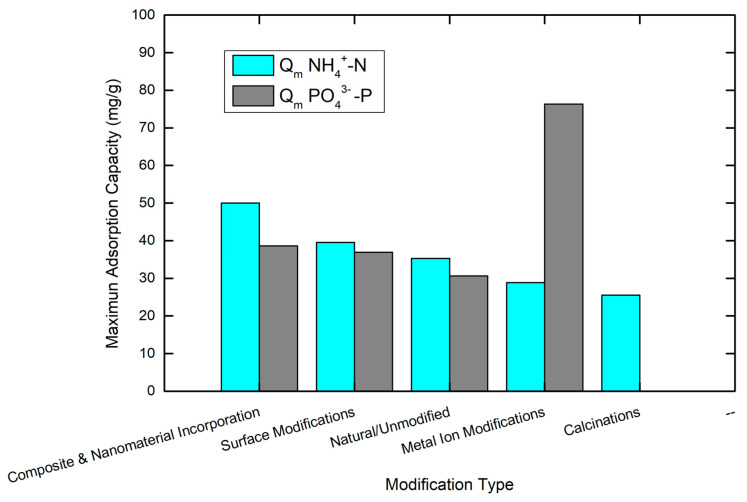
Visualization of the maximum adsorption capacities (mg/g) of both NH_4_^+^-N and PO_4_^3−^-P of the compared data presented in Table 2. The graph was created in Origin Lab (version 9).

The vast performance differences compared to natural zeolites from across a range of procurement locations continue to demonstrate the need to develop adequate characterization of source materials to develop potential modified inert-structure, high-performance, solid-phase removal materials. Further advantages may also exist in recognizing the primary local mineralogy with respect to the site’s water chemistry that the intended aquifer represents, so even undertaking localized optimization may prove to be a more cost-efficient approach compared to more universal modification methods. For visualization purposes, Figure 7 presents the data for the modification category.

Nonetheless, absolute comparison among different datasets is hard to obtain, since adsorption efficiency results are highly linked to site-specific variables, including initial nutrient loadings, hydraulic retention durations, and zeolite regeneration intervals. In batch and column experiments, equilibrium concentrations after natural and modified zeolites’ adsorption demonstrated that these adsorbents can effectively mitigate eutrophication when implemented at scale. Subsequently, to no surprise, pilot-scale applications will further deviate since they are subject to dynamic field conditions.

This review highlights the diverse range of zeolite modification schemes and the performance variabilities with respect to the uptake of nitrogen and phosphorus species. Many representative high-end materials that form the golden standard of zeolite-based water treatment technologies were discussed (e.g., Mg-ZIF nanosheets, lanthanum modified zeolites). Yet the complexity required to subsequently generate high-performance materials provides uncertainties with respect to their cost, scalability, and, thus, subsequent performance durability and performance stability over time when encountering operational conditions.

**Table 2:** Scientific literature comparison of zeolites’ ammonium and orthophosphate adsorption capacity.

Citation	Pilot/Lab/Field Study	Adsorption Capacity of NH_4_^+^-N (mg/g)	Adsorption Capacity of PO_4_^3−^-P (mg/g)	Adsorbent Dosage (mg/L)	PO_4_^3^-P Aquatic Solution (mg/L)	NH_4_^+^-N Aquatic Solution (mg/L)	HRT (h)	Origin	Particle Size	pH	Zeolite Form
[49]	Lab	21.3		1000		1–5000		Germany	1–2.5 mm 8–16 mm 16–32 mm	7	Natural zeolite (CLP85+)
[67]	Lab	7.64	0.86	1000	0–40	0–30	2			4–9	Lanthanum hydroxide (La-F4A) 4AZ
[8]	Lab	4.23	55.68	7000	5–100	5–100		China	Powder	6.35	EL-MNP@zeolite
[24]	Lab		28.9	400	0.05–5		3	Bulgaria	-	7.2	BePhos^TM^ (BFeLaHA)
[32]	Lab		1.54	3600			3	Ethiopia			Z-Al/Cu
[40]	Lab	36.87	36.88	100	0.1–100	0.1–100	24	Slovakia	<0.0105	7	ZeoPhos (ZCaFeHA)
[80]	Pilot			1000	0.1–10				0.5–1 cm		Lanthanum/aluminum hydroxide zeolite (LAH-Z)
[69]	Lab	16.6	6.62	2000	20–500	20–500	6	-	-	7	NaOH-activated and lanthanum-impregnated zeolite (NLZ)

### 4.5. The Influence of Environmental Factors

Environmental variables significantly affect the adsorption efficiency and capacity of ammonium (NH_4_^+^) and orthophosphate PO_4_^3−^ ions from aquatic systems, particularly with respect to eutrophication management of modified zeolites. These environmental factors include the ionic competition, Dissolved Organic Matter (DOM) competition, and the potential of leaching agents depending on zeolite modification, among others.

Comparing the recent scientific literature, studies show different cation orders for a competitive effect of ammonium adsorption. For example, Eberle et al. reported the competitive cation adsorption series K^+^ > Na^+^ > Mg^2+^ > Ca^2+^ [49], while others report that the competitive cation order is Na^+^ > K^+^ > Ca^2+^ > Mg^2+^or Ca^2+^ > K^+^ > Na^+^ > Mg^2+^. These discrepancies may be the result of different experimental conditions (different initial ion concentrations, pH, type of adsorbent, zeolite framework or exchange sites, temperature, etc.). Also, differences in zeolite modification processes, which may change the surface charge, the pore structure, and the ion exchange capacity of the zeolite, influence the strength of interaction of the various cations with the sites of adsorption. In general, the presence of competitive ions (particularly divalent ions) in real water matrices can dramatically decrease the effectiveness of ammonium adsorption in actual applications.

Moreover, the orthophosphate ion adsorption capacity of modified zeolites is decreased in the presence of divalent anions such as SO_4_^2−^ or HCO_3_^−^ [14,77]. However, lanthanum-modified zeolites are very selective, having practically no interference from any anions, such as Cl^−^ and NO_3_^−^, in the adsorption mechanism of orthophosphate [29,67]. Alternatively, Mg^2+^ and Ca^2+^ may also help facilitate the orthophosphate adsorption process from Fe/Al-modified zeolites due to Ca−P precipitation [77,81].

Furthermore, recent studies report that the Dissolved Organic Matter (DOM) present in freshwater, mostly through agricultural runoff, influences orthophosphate adsorption. In particular, DOM can negatively impact orthophosphate adsorption by competing for the active sites of the metal-modified zeolites [82].

The potential leaching of modifying agents is a serious environmental problem due to inhibited eco-toxicity concerns. La-modified zeolites show negligible La release at pH above 4.0 [29,78]. However, strong alkaline desorption (using NaOH) can damage the active surface, knocking the modifying agent, such as La or Fe, from the adsorbent surface by precipitation or detachment [67]. Synthesis processes that employ strong acids can result in the leaching of Al from the zeolite framework [83]. In addition, the use of natural compounds, has been suggested by some workers because of the lower endogenous risk of eco-toxicity compared with La or Zr compounds [77].

Unfortunately, it is rare to find true large-scale in situ and whole-lake pilots to address the multivariable phenomenon of eutrophication. Most in situ research is based on core tests of sediment capping/amendment or tank/column analogs using actual lake water. A study of Al-Zr-modified zeolite (AZMZ) [84] in bench/mesocosm capping tests aimed to mimic in-lake applications, whose adsorption capacity, measured against sediment–water nutrient flux, reached 8.40 and 3.10 mg/g for NH_4_^+^-N and PO_4_^3−^-P, respectively, for batch studies. The results for the mesocosm applications achieved 89% of the adsorption mentioned above in the capping studies. A similar quantitative study was conducted for Zr-modified zeolite applied at the sediment–water interface. The Zr-modified fly-ash zeolite was tested for both idealized exposure and potentially in the field with actual lake water [33]. It was observed that the potential of P-loadings reached up to 3.015 mg-P/g at pH 7 (3 g/L dose), whereas in the sediment–water interface the capacity fell to ~0.186 mg-P/g. In general, field pilot tests have shown substantially lower fluxes of NH_4_-N and P from sediments (often large % changes in fluxes), while laboratory isotherm tests report very wide ranges of adsorption capacities: phosphate 1–50+ mg P·g^−1^ (pristine zeolite at the low end, lanthanum/Fe/other modifications at the high end) and ammonium ~1–25 mg N·g^−1^, depending on modification and conditions [85].

## 5. Conclusions

Eutrophication has been identified as a rising global concern for aquatic ecosystems, and therefore, researchers have increased their efforts towards finding efficient nutrient removal technologies. Zeolite materials, with their unique crystalline aluminosilicate structure and excellent ion exchange ability, have been identified as a suitable material for concurrent removal of ammonium nitrogen and orthophosphate phosphorus from eutrophic freshwater bodies.

This review highlights that zeolites are a promising solution for the management of eutrophication, but it is essential to have (i) standard methods for measuring ion exchange capacity and (ii) large-field pilot tests in natural freshwater systems. Filling these gaps will enable a more thorough assessment of zeolites as sustainable materials for managing nutrients in aquatic ecosystems. Furthermore, nutrient-rich zeolites collected from eutrophic waters could be used for nutrient-rich products, e.g., slow-release fertilizers in agriculture, thus allowing for some level of recovery from nutrient pollution for a resource. Incorporating a circular economy in this manner can enhance the economic justification of using zeolites while also meeting larger sustainability objectives to close nutrient loops. Most importantly, integrating zeolite-based adsorption with practices of the circular economy can be moved from just immediate mitigation to long-term ecological restoration of eutrophic waterbodies while reducing future risk of nutrient pollution.

At the same time, very few studies have examined zeolite performance under environmentally relevant conditions, and rarely do studies look at long-term stability and regeneration capacity of modified zeolites. Additionally, nutrient removal is often conducted in isolation, so the complex ionic interactions that exist in eutrophic systems are overlooked. Solving these knowledge gaps will be critical to moving zeolite-based strategies forward from the lab to field scale.

Future studies should establish inexpensive synthetic processes that are designed to create high-value composites and optimize dual-function properties with systematic modifications. The long-term functional stability of modified zeolites should be studied under realistic water treatment applications and from a circular economy perspective. Research on zeolite-based nutrient removal has mainly focused on adsorption efficiency, with limited information available concerning ionic competition, regeneration cycles, and the potential leaching of modifying agents. Future studies must thoroughly examine these operational and environmental aspects to facilitate the dependable application of laboratory findings to field-scale eutrophication management. Advanced characterization techniques would aid in the performance assessment to create a holistic roadmap of next-generation zeolite materials that can be applied to specific water treatment applications, recovering nutrients, and reintroduction into the environment.

## Figures and Tables

**Table 1:** Comparative studies of zeolite particle size and ammonium adsorption capacity.

Zeolite Form	Particle Size (mm)	Ammonium Ion Adsorption Capacity (mg/g)	Size Category	Citation
Romanian natural zeolite	0.5–1.25 mm	12.34	Medium (0.5–2 mm)	[60]
	1.25–3.0 mm	11.4	Large (2–10 mm)	
	>3.0 mm	10.46	Large (2–10 mm)	
CLP85+ (clinoptilolite)	1–2.5	31.43	Medium (0.5–2 mm)	[49]
	8–16	21.3	Very large (>10 mm)	
	16–32	16.21	Very large (>10 mm)	
Micro-sized zeolite particles (ZPs)	0.0048	60.6	Ultra-fine (<0.1 mm)	[64]
PAZ hydrogel beads (PVA–alginate matrix with entrapped ZPs)	4.04	28.2	Ultra-fine (<0.1 mm)	

## Data Availability

No new data were created or analyzed in this study. Data sharing is not applicable to this article.
